# Occurrence and Genomic Characterization of Two MCR-1-Producing Escherichia coli Isolates from the Same Mink Farmer

**DOI:** 10.1128/mSphere.00602-19

**Published:** 2019-11-06

**Authors:** Beiwen Zheng, Hao Xu, Chen Huang, Xiao Yu, Lihua Guo, Huiming Han, Jing Zhang, Xiawei Jiang, Chunlei Chen, Yonghong Xiao

**Affiliations:** a Collaborative Innovation Center for Diagnosis and Treatment of Infectious Diseases, State Key Laboratory for Diagnosis and Treatment of Infectious Diseases, The First Affiliated Hospital, School of Medicine, Zhejiang University, Hangzhou, China; b Department of Respiratory Medicine, Lihuili Hospital, Ningbo Medical Center, Ningbo, China; c Medical College of Beihua University, Jilin, China; d Department of Pulmonary and Critical Care Medicine, Guangdong General Hospital, Guangzhou, China; e College of Basic Medical Sciences, Zhejiang Chinese Medical University, Hangzhou, China; Escola Paulista de Medicina/Universidade Federal de São Paulo

**Keywords:** farmer, MCR-1.12, ESBLs, PacBio, coexistence, mink

## Abstract

The spread of colistin resistance gene *mcr-1* at the animal-human interface remains largely unknown. This work aimed to investigate the molecular characteristics of two extended-spectrum-β-lactamase (ESBL)-producing Escherichia coli strains with *mcr-1*, i.e., strains H8 and H9, isolated from the same mink farmer. In this study, five *mcr-*positive E. coli strains were isolated from the mink farm. Pulsed-field gel electrophoresis (PFGE) and multilocus sequence typing (MLST) identified two genetically unrelated MCR-1 producers (H8 and H9) from the same farmer and two clonally related MCR-1-positive isolates (M5 and M6) from two different mink samples. Additionally, a *mcr-1* variant, designated *mcr-1.12*, was identified in isolate M4. MIC determination revealed that all of the MCR-producing strains exhibited multiresistant phenotypes but showed susceptibility to imipenem, meropenem, amikacin, and tigecycline. Replicon typing showed that *mcr-1* was associated with IncHI2 plasmids in 4 cases, while the gene was located on an IncI2 plasmid in 1 case. PacBio sequencing and plasmid analysis confirmed that the *mcr*-*1* gene was located on an ∼204-kb IncHI2 plasmid in H8 and was carried by an ∼61-kb IncI2 plasmid in H9. To our knowledge, this work represents the first report of the occurrence of MCR-producing isolates from mink. Moreover, our report also describes the coexistence of two different MCR-1 producers in the same farmer. It highlights that fur farms can be reservoirs of *mcr-1* genes. The identification of *mcr*-carrying plasmids on a fur farm is of potential public health importance, as it suggests that *mcr* is widespread in the animal husbandry industry.

**IMPORTANCE** Colistin resistance is a real threat for both human and animal health. The mobile colistin resistance gene *mcr* has contributed to the persistence and transmission of colistin resistance at the interfaces of animals, humans, and ecosystems. Although *mcr* genes have usually been recovered from food animals, patients, and healthy humans, transmission of *mcr* genes at the animal-human interface remains largely unknown. This was the first study to isolate and characterize MCR-producing isolates from mink, as well as to report the coexistence of two different MCR-1 producers in the same farmer. The characterization and analysis of two MCR-1-producing E. coli isolates may have important implications for comprehension of the transmission dynamics of these bacteria. We emphasize the importance of improved multisectorial surveillance of colistin-resistant E. coli in this region.

## Abstract (precis)

Colistin resistance is a real threat for both human and animal health. The mobile colistin resistance gene *mcr* has contributed to the persistence and transmission of colistin resistance at the interfaces of animals, humans, and ecosystems. Although *mcr* genes have usually been recovered from food animals, patients, and healthy humans, transmission of *mcr* genes at the animal-human interface remains largely unknown. This was the first study to isolate and characterize MCR-producing isolates from mink, as well as to report the coexistence of two different MCR-1 producers in the same farmer. The characterization and analysis of two MCR-1-producing E. coli isolates may have important implications for comprehension of the transmission dynamics of these bacteria. We emphasize the importance of improved multisectorial surveillance of colistin-resistant E. coli in this region.

## OBSERVATION

Colistin is one of the most critically important antimicrobials and is considered a drug of last resort for the treatment of infections caused by multidrug-resistant pathogens. The transmissible colistin resistance gene *mcr-1* was first discovered in food animals and humans in 2015 (1). Since then, this gene has been detected in pets, wild birds, and environmental samples from various sources (2). Interestingly, a previous study reported the possibility that extended-spectrum β-lactamases (ESBLs), carbapenemase enzymes, and MCR-1 have coexisted since the 1980s (3). The cooccurrence of carbapenemase genes or ESBL genes and *mcr-1* in the same isolate is of concern because it has the potential to lead to pan-drug resistance (4).

Previous studies have confirmed the spread of *mcr* genes mainly via epidemic plasmids (IncI2, IncHI2, IncP, IncX4, IncFI, and IncFIB) of various sizes (58 to 251 kb) (5). The acquisition of the *mcr-1* gene has been investigated extensively, and diverse genetic contexts surrounding *mcr-1* genes have been discovered (6). However, comprehensive information regarding the prevalence of *mcr-1* in isolates from fur-bearing animals and the spread of colistin resistance at the animal-human interface remains limited.

We performed a survey of fecal isolates from two adjacent farms (a fur farm and a household farm) located in Shandong Province, China, in June 2016. A total of 20 samples from fur animals, 10 from chicken, 5 from pigs, and 10 from farmers were collected. One fecal sample was collected from each farmer and animal. Fecal samples were collected using sterile swabs and stored in sterile tubes. Subsequently, samples were placed on ice, transported to the laboratory, and processed within 24 h after collection.

Fecal samples were cultured on MacConkey agar plates supplemented with 2 mg/liter cefotaxime at 37°C for 24 h under aerobic conditions to recover potential ESBL-producing isolates. The MICs were determined by the agar dilution method and interpreted according to CLSI standards (7), and EUCAST breakpoints for tigecycline, colistin, and polymyxin B were applied. Escherichia coli strain ATCC 25922 was used as a quality control.

Each isolate was tested for the presence of genes encoding ESBLs (8, 9) and MCR variants via PCR and sequencing (see Table S1 in the supplemental material). The clonal relatedness of the E. coli isolates was assessed using pulsed-field gel electrophoresis (PFGE) (8) and multilocus sequence typing (MLST) (http://enterobase.warwick.ac.uk/species/ecoli/allele_st_search).

10.1128/mSphere.00602-19.3 TABLE S1 PCR primers used in this study. Download Table S1, DOCX file, 0.03 MB. Copyright © 2019 Zheng et al. 2019 Zheng et al. This content is distributed under the terms of the Creative Commons Attribution 4.0 International license.

MCR-positive isolates were characterized by conjugation experiments to assess their ability to transfer colistin resistance (10). Plasmid sizes were determined using S1-PFGE and Southern blotting methods as previously described (8). The identification of the plasmid incompatibility (Inc) group replicon types was performed by multiplex PCR, as described previously (11). The circular image representing comparisons of multiple plasmids was generated by the BLAST Ring Image Generator (BRIG) (12).

Genomic DNA was extracted using an Omega bacterial DNA kit (Omega Bio-tek, Norcross, CA, USA). Illumina sequencing was performed on MCR-1 variant isolate M4 and on MCR-1-producing isolates from the same farmer (H8 and H9) with the HiSeq 4000-PE150 platform (Illumina, San Diego, CA, USA). Illumina sequencing libraries were prepared using standardized protocols (6, 13). To obtain the complete sequences of pMCR-H8 and pMCR-H9, isolates H8 and H9 were further subjected to single-molecule real-time (SMRT) DNA sequencing on a PacBio RS II platform (Pacific Biosciences, CA, USA). The complete genome sequences for E. coli H8 and H9 were created by combining Illumina sequencing reads with PacBio sequencing reads, using Unicycler (14). PlasmidSPAdes was used to produce plasmid sequences from whole-genome-sequencing (WGS) data (http://spades.bioinf.spbau.ru/plasmidSPAdes/). Additionally, *in silico* sequence analyses of multilocus sequencing typing (MLST) data, antimicrobial resistance genes, and plasmid replicon typing were performed using online tools (http://www.genomicepidemiology.org/).

**FIG 1:**
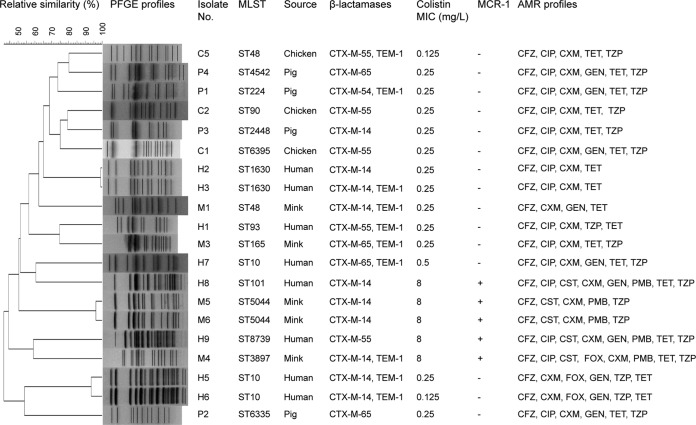
Summary of the molecular epidemiological characteristics of ESBL-producing E. coli strains identified from adjacent farms in China, 2016. The dendrogram of PFGE patterns was constructed using BioNumerics v6.6 with unweighted pair group method using average linkages (UPGMA) clustering. AMK, amikacin; CFZ, cefazolin; CIP, ciprofloxacin; CST, colistin; CXM, cefuroxime; FOX, cefoxitin; GEN, gentamicin; IMP, imipenem; MEM, meropenem; PMB, polymyxin B; PTZ, piperacillin-tazobactam; TET, tetracycline; TGC, tigecycline; ESBL, extended-spectrum β-lactamase; Neg, negative; Pos, positive. MICs were determined according to CLSI standards. The EUCAST breakpoints for tigecycline, colistin, and polymyxin B were applied.

Overall, 20 ESBL-producing E. coli isolates were identified from human and animal fecal samples (Fig. 1). We detected *bla*_CTX-M_ genes in all isolates, including *bla*_CTX-M-14_ (*n* = 10), *bla*_CTX-M-65_ (*n* = 4), and *bla*_CTX-M-54_ (*n* = 1). The remaining ESBL isolates belonged to the *bla*_CTX-M-55_ group (*n* = 5). We further identified 5 (22.7%) *mcr*-positive E. coli isolates from the fur farm (Fig. 1). Among them, 3 were isolated from mink samples, while the remaining 2 were detected in the same farmer. DNA sequencing revealed that four isolates carried the *mcr-1* gene, while isolate M4 harbored a new *mcr* variant. The *mcr-1.12* gene, as designated here, carries a missense mutation at position 48 (T→G), which results in a Phe16-to-Leu substitution. MIC determination revealed that all of the MCR-producing strains exhibited multiresistant phenotypes but showed susceptibility to imipenem, meropenem, amikacin, and tigecycline (Fig. 1).

Genetic diversity was observed in the PFGE profiles, and no obvious major pulsotypes were found via PFGE analysis. Isolates H2 and H3, isolates M5 and M6, and isolates H2 and H3 shared high homology, respectively (Fig. 1). MLST of the 20 E. coli isolates identified 15 unique sequence types (STs). ST10 (*n* = 3) was the predominant ST among the isolates, with all ST10 isolates being recovered from farm workers (Fig. 1). Four different STs were detected among the 5 E. coli isolates carrying *mcr* genes. Of note, two MCR-1-positive isolates (H8 and H9) from the same fur animal farm worker belonged to ST101 and ST6390, respectively. Transconjugation experiments, replicon typing, and S1-PFGE demonstrated the cooccurrence of *mcr-1* and *bla*_CTX-M-9_ group genes on the three IncHI2 plasmids (pMCR-M4, pMCR-M5, and pMCR-M6) of ∼218 kb and one IncHI2 plasmid (pMCR-H8) of ∼206 kb (see Fig. S1 in the supplemental material). Transconjugants from H8 isolates exhibited elevated colistin, ampicillin, cefazolin, and ceftriaxone MICs; by contrast, transconjugants from H9 showed elevated colistin MICs only (Table S2).

10.1128/mSphere.00602-19.1 FIG S1 Plasmid analysis of *mcr-1*-carrying plasmids. (A) Plasmid profiles of five MCR-1-producing and ESBL-producing Escherichia coli isolates determined using restriction enzyme S1, with Salmonella enterica serovar Braenderup as a molecular mass marker. (B) Southern blot hybridization with an *mcr*-specific probe and a *bla*_CTX-M-9_-specific probe. Lane 1, isolate M4; lane 2, isolate M5; lane 3, isolate M6; lane 4, isolate H8; lane 5, isolate H9. Smears show Southern blot analysis of plasmid DNA with probes specific to *mcr* and *bla*_CTX-M-9_ group genes. Download FIG S1, TIF file, 2.7 MB. Copyright © 2019 Zheng et al. 2019 Zheng et al. This content is distributed under the terms of the Creative Commons Attribution 4.0 International license.

10.1128/mSphere.00602-19.4 TABLE S2 Drug MIC values determined for two MCR-1-producing isolates and their recipient strains. Download Table S2, DOCX file, 0.02 MB. Copyright © 2019 Zheng et al. 2019 Zheng et al. This content is distributed under the terms of the Creative Commons Attribution 4.0 International license.

**FIG 2:**
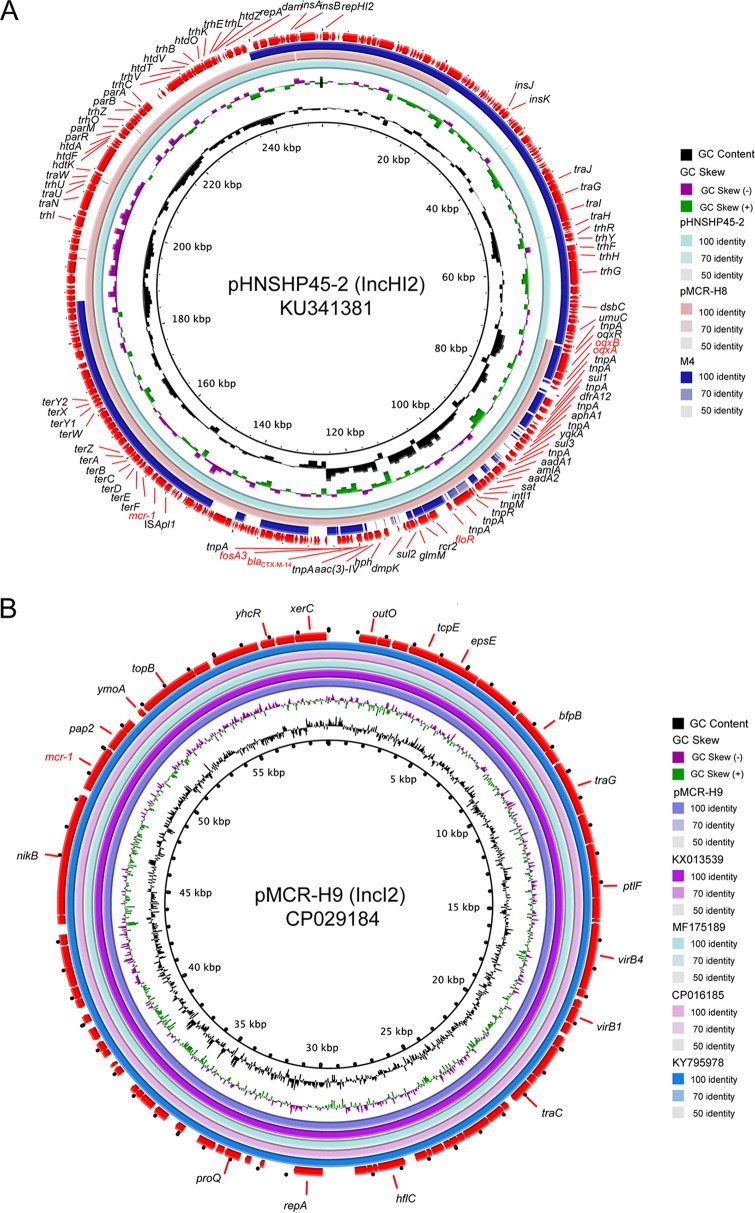
Complete sequences of two *mcr-1*-carrying plasmids detected in the same fur farmer. (A) Alignment of plasmid sequence of pMCR-H8 with IncHI2 plasmids pHNSHP45-2 (KU341381) and pMCR-M4. Sequence of pM4MCR contig was generated by plasmidSPAdes. (B) Comparison of pMCR-H9 with other IncI2 plasmids harboring *mcr-1* gene. The following plasmids were included in the comparison: pBA77-MCR-1 (KX013539), pColR598_2 (MF175189), pEC5-1 (CP016185), and pJIE3685-1 (KY795978). Circles (from inside to outside) denote the GC content, the GC skew, and the open reading frames (ORFs) in both DNA strands. Block arrows represent coding sequences and indicate the direction of transcription. Antimicrobial resistance genes are highlighted in red. Arrow size is proportional to gene length. The circular image representing comparisons of multiple plasmids was generated by BRIG.

The complete sequence of pMCR-H8 harboring the *mcr-1* gene was 203,941 bp (Fig. 2A; see also Table S3). Plasmid comparisons based on full-plasmid BLAST query revealed that pMCR-H8 was closely related to plasmid pHNSHP45-2 (GenBank no. KU341381), which was described in the original report of the discovery of *mcr-1* (15). Annotations showed that pMCR-H8 also carried genes conferring resistance to crucially important antibiotics for human treatment, including cephalosporin (*bla*_CTX-M-14_), florfenicol (*floR*), fosfomycin (*fosA3*), and quinolone (*oqxAB*) (Fig. 2A; see also Table S3). In contrast, pMCR-H9 was found to be a 60,942-bp IncI2 plasmid. The overall structure of pMCR-H9 was identical to that of various IncI2-type plasmids (Fig. 2B), including pJIE3685-1, identified from a human urine E. coli isolate collected in Australia (16), and pColR598_2, recovered from a human isolate collected in Switzerland (17). Interestingly, pMCR-H8 and pMCR-M4 exhibited low sequence identity (Fig. 2A), although they are similar in size (Fig. S1). These results suggest the genetic diversity of *mcr-1*-carrying plasmids from mink and human origin and the flexibility of *mcr-1* transmission between animal and human.

10.1128/mSphere.00602-19.5 TABLE S3 Genome features of MCR-1-producing E. coli isolates H8 and H9. Download Table S3, DOCX file, 0.02 MB. Copyright © 2019 Zheng et al. 2019 Zheng et al. This content is distributed under the terms of the Creative Commons Attribution 4.0 International license.

In this work, the *mcr-1* gene was found in three genetic contexts, including *mcr-1 pap2*, IS*Apl1 mcr-1 pap2*, and IS*Apl1 mcr-1 pap2* IS*Apl1*. The complete sequence of pMCR-H8 revealed the presence of IS*Apl1 mcr-1 pap2*, while IS*Apl1* was absent in pMCR-H9 (Fig. 2). We further identified an IncHI2 plasmid contig carrying the *mcr-1.12* gene from isolate M4 using the plasmidSPAdes assembler with high coverage. Querying this ∼43-kb-length contig against the NCBI nr/nt sequence database revealed 100% sequence homology to annotated *mcr-1*-positive IncHI2 plasmid pHNSHP45-2 (GenBank: KU341381) (Fig. 2A). A typical structure surrounding the *mcr-1* gene (IS*Apl1 mcr-1.12 pap2* IS*Apl1*) was observed in the plasmid contig (Fig. S2).

10.1128/mSphere.00602-19.2 FIG S2 Alignment of contig-containing conjugative IncHI2 plasmid pM4MCR with sequence of pHNSHP45-2. The sequence of the pM4MCR contig was generated by plasmidSPAdes. Block arrows represent coding sequences (red arrow, *mcr-1.12*; green arrows, IS*apl1*; cyan arrow, *pap2*; pink arrow, HNH endonuclease-encoding gene; gray arrows, other coding DNA sequences [CDS]) and indicate the direction of transcription. Arrow size is proportional to gene length. The gray areas between pM4MCR and pHNSHP45-2 indicate nucleotide identity of >99% according to BLASTN. The gaps in pM4 MCR represent missing bases compared to the reference plasmid. The insertion sequence (IS) is represented as an open box in which the terminal inverted repeats (IRs) are shown as black boxes labelled IRL (left inverted repeat) and IRR (right inverted repeat). Download FIG S2, TIF file, 0.3 MB. Copyright © 2019 Zheng et al. 2019 Zheng et al. This content is distributed under the terms of the Creative Commons Attribution 4.0 International license.

Previous studies have shown that farm workers have a higher rate of carriage of ESBL/AmpC-producing E. coli than the general population (18). The cooccurrence of two different MCR-1-producing isolates in the same farmer indicates that individuals in direct contact with minks are at potential risk for carrying MCR-1 producers, although the magnitude of this risk remains to be elucidated.

Colistin is widespread used in animals but rarely used on mink breeding farms in China (1, 19). Our retrospective questionnaire also revealed that only flavomycin was used for growth promotion on the fur farm, while no history of usage of polymyxins was reported from the farmers. Recently, Wu et al. reported the rapid rise in carriage of the ESBL and *mcr-1* genes in E. coli of chicken origin in Shandong Province (20). Previous investigations also found that MCR variants were widespread among samples from farmers (21, 22). We hypothesized that the introduction of *mcr-1*-positive bacteria in fur farms had occurred via the food chain-based dissemination pathway, since the farmer reported the breed history of mink fed with chicken bones. Thus far, only limited reports have described the patterns of antimicrobial use and antimicrobial-resistant bacteria among mink (23, 24). The epidemiology of antimicrobial-resistant bacteria among mink and farm workers is largely unknown. Therefore, a “One Health” strategy for preventing the spread of colistin resistance at the human-mink-environment interface is essential.

The major types of *mcr-1*-carrying plasmids, including IncI2, IncHI2, IncX4, IncP, IncFI, and IncFIB plasmids of various sizes, have been identified in E. coli isolates from diverse hosts (5). In fact, *mcr-1*-carrying IncHI2/IncI2 plasmids were frequently identified from animal, human, and environmental samples in China (20, 25). Our data further confirmed that similar IncHI2 and IncI2 plasmids carrying *mcr-1* gene are prevalent in various sources in China.

In summary, we describe the emergence of MCR-1 producers in mink and the coexistence of two different MCR-1-producing E. coli isolates in the same farmer. Notwithstanding the limitations of this work, our findings indicate that *mcr-1*-carrying IncHI2/IncI2 plasmids are widely disseminated in China. Additional studies involving more fur animals and farm workers are urgently needed to assess the dissemination dynamics of *mcr* genes.

### Data availability.

Whole-genome shotgun BioProject data for the E. coli H8 and H9 isolates have been deposited in GenBank under BioProject accession no. PRJNA449899. The whole-genome sequence of the E. coli M4 isolate has been deposited in GenBank under accession no. MTRZ00000000. The *mcr-1.12* gene sequence has been deposited in GenBank under accession no. KY400027.
