# Supplementary material for: Occurrence and Genomic Characterization of Two MCR-1-Producing Escherichia coli Isolates from the Same Mink Farmer
Source: mSphere. 2019 Nov 6;4(6):e00602-19. doi: 10.1128/mSphere.00602-19 (PMC6835210; doi:10.1128/mSphere.00602-19)
Supplement: TABLE S1 [file mSphere.00602-19-st001.docx]

**Table S1**

| **Target gene** | **Sequence** | **Reference** |
| --- | --- | --- |
| *mcr-1* | 5ʹ-CGGTCAGTCCGTTTGTTC-3ʹ | 1 |
|  | 5ʹ-CTTGGTCGGTCTGTAGGG-3ʹ |  |
| *mcr-2* | 5ʹ-TGGTACAGCCCCTTTATT--3ʹ | 2 |
|  | 5ʹ-GCTTGAGATTGGGTTATGA-3ʹ |  |
| *mcr-3* | 5ʹ-TTGGCACTGTATTTTGCATTT-3ʹ | 3 |
|  | 5ʹ-TTAACGAAATTGGCTGGAACA-3ʹ |  |
| *mcr-4* | 5ʹ-ATTGGGATAGTCGCCTTTTT-3ʹ | 4 |
|  | 5ʹ-TTACAGCCAGAATCATTATCA-3ʹ |  |

1. Liu YY, Wang Y, Walsh TR, Yi LX, Zhang R, Spencer J, Doi Y, Tian G, Dong B, Huang X, Yu LF, Gu D, Ren H, Chen X, Lv L, He D, Zhou H, Liang Z, Liu JH, Shen J. 2016. Emergence of plasmid-mediated colistin resistance mechanism MCR-1 in animals and human beings in China: a microbiological and molecular biological study. Lancet Infect Dis 16:161-8.

2. Xavier BB, Lammens C, Ruhal R, Kumar-Singh S, Butaye P, Goossens H, Malhotra-Kumar S. 2016. Identification of a novel plasmid-mediated colistin-resistance gene, mcr-2, in Escherichia coli, Belgium, June 2016. Euro Surveill 21.

3. Yin W, Li H, Shen Y, Liu Z, Wang S, Shen Z, Zhang R, Walsh TR, Shen J, Wang Y. 2017. Novel Plasmid-Mediated Colistin Resistance Gene mcr-3 in Escherichia coli. MBio 8.

4. Carattoli A, Villa L, Feudi C, Curcio L, Orsini S, Luppi A, Pezzotti G, Magistrali CF. 2017. Novel plasmid-mediated colistin resistance mcr-4 gene in Salmonella and Escherichia coli, Italy 2013, Spain and Belgium, 2015 to 2016. Euro Surveill 22.
