# Supplementary figures and images for: Occurrence and Genomic Characterization of Two MCR-1-Producing Escherichia coli Isolates from the Same Mink Farmer
Source: mSphere. 2019 Nov 6;4(6):e00602-19. doi: 10.1128/mSphere.00602-19 (PMC6835210; doi:10.1128/mSphere.00602-19)

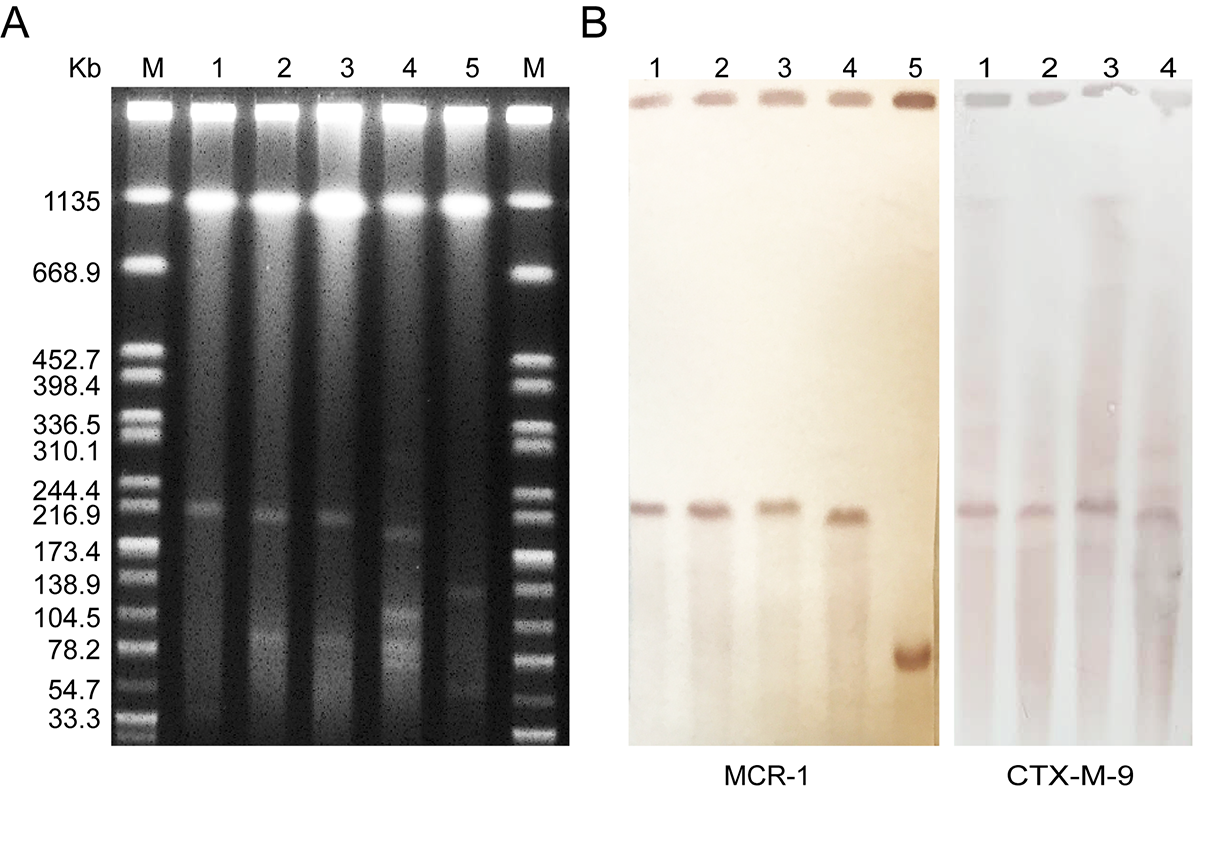

Supplement: FIG S1 [file mSphere.00602-19-sf001.tif]

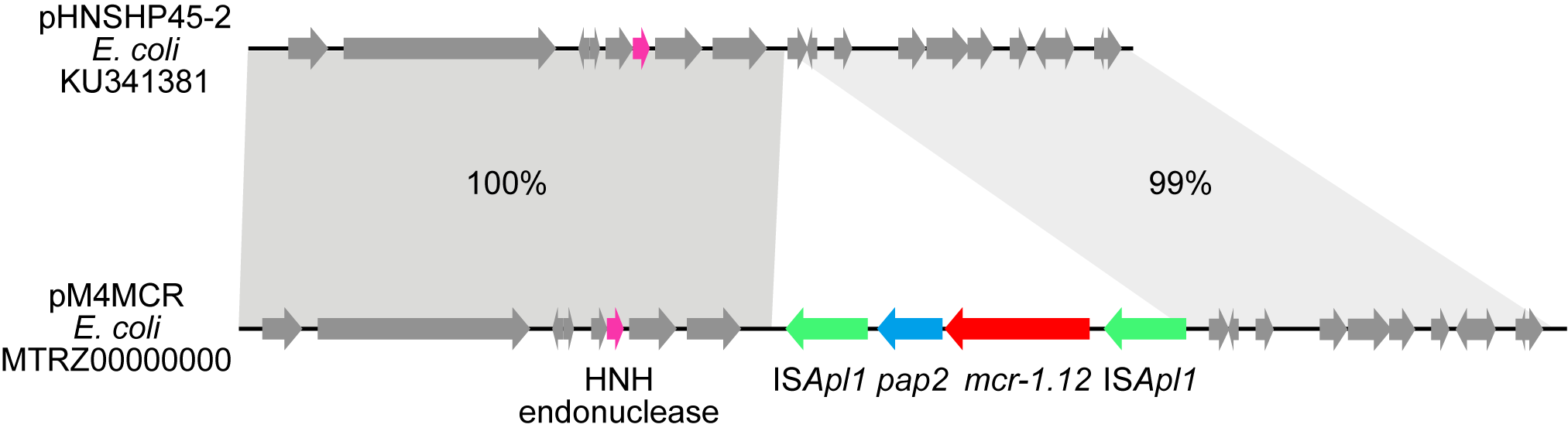

Supplement: FIG S2 [file mSphere.00602-19-sf002.tif]
