# Supplementary material for: Occurrence and Genomic Characterization of Two MCR-1-Producing Escherichia coli Isolates from the Same Mink Farmer
Source: mSphere. 2019 Nov 6;4(6):e00602-19. doi: 10.1128/mSphere.00602-19 (PMC6835210; doi:10.1128/mSphere.00602-19)
Supplement: TABLE S2 [file mSphere.00602-19-st002.docx]

**Table S2**

| Antibiotics | MIC (mg/liter) | | | | |
| --- | --- | --- | --- | --- | --- |
|  | *E. coli*  EC600 | *E. coli* H8 | *E. coli* TC  EC600: H8 | *E. coli*  H9 | *E. coli* TC  EC600: H9 |
| Imipenem | 0.25 | 1 | 1 | 1 | 1 |
| Ertapenem | 0.125 | 0.5 | 0.5 | 0.5 | 0.5 |
| Ampicillin | 8 | 32 | 32 | 32 | 8 |
| Cefazolin | 4 | 64 | 64 | 64 | 4 |
| Ceftriaxone | 1 | 64 | 64 | 64 | 1 |
| Cefepime | 1 | 1 | 2 | 8 | 1 |
| Aztreonam | 1 | 1 | 4 | 16 | 1 |
| Gentamicin | 1 | 16 | 16 | 8 | 1 |
| Tobramycin | 1 | 16 | 16 | 8 | 1 |
| Amikacin | 1 | 2 | 2 | 2 | 2 |
| Tigecycline | 0.5 | 0.5 | 0.5 | 0.5 | 0.5 |
| Colistin | 0.25 | 8 | 4 | 8 | 4 |
| Nitrofurantoin | 16 | 32 | 16 | 64 | 16 |
| Piperacillin-tazobactam | 4 | 4 | 4 | 4 | 4 |
| Trimethoprim-sulfamethoxazole | <20 | >320 | >320 | <20 | <20 |
| Amoxicillin-clavulanic acid | 4 | 4 | 16 | 4 | 4 |
| Levofloxacin | 0.5 | 8 | 1 | 8 | 0.5 |
| Ciprofloxacin | 0.25 | 4 | 1 | 4 | 0.25 |

Antibacterial drug susceptibility was determined by microdilution method according to the guidelines of the Clinical and Laboratory Standards Institute.
