# Supplementary material for: Occurrence and Genomic Characterization of Two MCR-1-Producing Escherichia coli Isolates from the Same Mink Farmer
Source: mSphere. 2019 Nov 6;4(6):e00602-19. doi: 10.1128/mSphere.00602-19 (PMC6835210; doi:10.1128/mSphere.00602-19)
Supplement: TABLE S3 [file mSphere.00602-19-st003.docx]

**Table S3.** Genome features of MCR-1-producing *E. coli* isolates H8 and H9

| **Isolate** | **Replicon** | **Inc type** | **Size (bp)** | **GC (%)** | **Accession no.** | **Antimicrobial resistance genes*** |
| --- | --- | --- | --- | --- | --- | --- |
| H8 | chromosome | *oriC* | 4,742,904 | 50.4 | CP029212 | *mdfA* |
|  | pMCR-H8 | IncHI2 | 203,941 | 46.9 | CP029215 | *aac3-IV, aph3'-Ia, aph4-Ia, aadA1, aadA2, bla*_CTX-M-14_*, mcr-1, oqxA, oqxB, fosA3, floR, cmlA1, sul1, sul2, sul3, dfrA12* |
|  | P2 | IncI1 | 124,242 | 51.3 | CP029213 | ND |
|  | P3 | IncFIA | 95,649 | 51.6 | CP029214 | *tetA* |
| H9 | chromosome | *oriC* | 4,722,506 | 50.9 | CP029180 | *aph6-Id, aph3''-Ib, bla*_CTX-M-55_*, mdfA, floR, sul2, tetA* |
|  | pMCR-H9 | IncI2 | 60,942 | 42.4 | CP029184 | *mcr-1* |
|  | P1 | p0111 | 145,524 | 49.2 | CP029181 | *aadA1, oqxA, oqxB, tetA, dfrA1* |
|  | P3 | untypeable | 31,250 | 50.9 | CP029182 | *aac3-IId, aph3'-IIa, mphA* |
|  | P4 | Col(MG828) | 1,551 | 51.6 | CP029183 | ND |
